# Systematic Complex Haploinsufficiency-Based Genetic Analysis of *Candida albicans* Transcription Factors: Tools and Applications to Virulence-Associated Phenotypes

**DOI:** 10.1534/g3.117.300515

**Published:** 2018-02-22

**Authors:** Virginia E. Glazier, Thomas Murante, Kristy Koselny, Daniel Murante, Marisol Esqueda, Gina A. Wall, Melanie Wellington, Chiung-Yu Hung, Anuj Kumar, Damian J. Krysan

**Affiliations:** * Department of Pediatrics, University of Rochester Medical Center, Rochester, New York, 14642; † Department of Biology and South Texas Center for Emerging Infectious Diseases, University of Texas, San Antonio, TX 78249, USA; ‡ Department of Molecular, Cellular and Developmental Biology, University of Michigan 48109

**Keywords:** *Candida albicans*, haploinsufficiency, complex haploinsufficiency, hyphal morphogenesis, biofilm formation, disseminated candidiasis

## Abstract

Genetic interaction analysis is a powerful approach to the study of complex biological processes that are dependent on multiple genes. Because of the largely diploid nature of the human fungal pathogen *Candida albicans*, genetic interaction analysis has been limited to a small number of large-scale screens and a handful for gene-by-gene studies. Complex haploinsufficiency, which occurs when a strain containing two heterozygous mutations at distinct loci shows a phenotype that is distinct from either of the corresponding single heterozygous mutants, is an expedient approach to genetic interactions analysis in diploid organisms. Here, we describe the construction of a barcoded-library of 133 heterozygous TF deletion mutants and deletion cassettes for designed to facilitate complex haploinsufficiency-based genetic interaction studies of the TF networks in *C. albicans*. We have characterized the phenotypes of these heterozygous mutants under a broad range of *in vitro* conditions using both agar-plate and pooled signature tag-based assays. Consistent with previous studies, haploinsufficiency is relative uncommon. In contrast, a set of 12 TFs enriched in mutants with a role in adhesion were found to have altered competitive fitness at early time points in a murine model of disseminated candidiasis. Finally, we characterized the genetic interactions of a set of biofilm related TFs in the first two steps of biofilm formation, adherence and filamentation of adherent cells. The genetic interaction networks at each stage of biofilm formation are significantly different indicating that the network is not static but dynamic.

*Candida albicans* is one of the most important fungal pathogens of humans and causes disease in both immunocompetent and immunocompromised individuals (Köhler *et al.* 2014). The application of genetic analysis to *C. albicans* has been, and will remain, crucial to understanding pathogenesis, host-fungus interactions, and mechanisms of drug action (Xu *et al.* 2014). Over the past twenty-five years, most of the modern tools and techniques of molecular genetics developed in other systems have been applied to the study of *C. albicans* (Hernday *et al.* 2010). These include the development of recyclable genetic markers, PCR-based cassette generation, large-scale collections of mutants and inducible alleles, and, most recently, CRISPR/Cas9-based genetic editing (Vyas *et al.* 2015).

One area of *C. albicans* genetic analysis that has not been extensively developed compared to other systems is genetic interaction analysis. Genetic interaction analysis allows the identification and characterization of networks of genes whose products work together to regulate and/or mediate a common cellular process. Genetic interaction analysis is currently most highly developed in the model yeast *Saccharomyces cerevisiae* (Tong *et al.* 2004). Through clever application of genetics and high throughput biology, the facile mating cycle of *S. cerevisiae* has been harnessed to generate genetic interaction networks encompassing ∼90% of its genes and include ∼23 million double mutants (Costanzo *et al.* 2016). Although *C. albicans* is arguably the most mature genetic system for pathogenic fungi, genetic interaction analysis has remained significantly under-developed.

One of the barriers to genetic interaction analysis in *C. albicans* is that it is primarily a diploid organism without a traditional sexual cycle (Hickman *et al.* 2013). Typically, genetic interactions are studied by generating strains that contain null mutants of the two genes of interest. The phenotypes of the resulting double mutants are then compared with the single mutants. For *S. cerevisiae* and other haploid organisms, the construction of the requisite double null mutant involves two gene deletion events. In the diploid *C. albicans*, however, four discrete gene deletions are required to generate the mutants at two loci. Not surprisingly, multiple null mutants have been constructed infrequently in *C. albicans* and, until recently, no systematic approaches to studying large numbers of double mutants have been reported. Of course, the recent applications of CRISPR/Cas9 to *C. albicans* are likely to increase the number of multiple homozygous gene deletions that are studied (Nguyen *et al.* 2017).

Double homozygous mutants, however, will not be applicable to, or appropriate for, all systems. An alternative strategy for genetic interaction analysis in diploids is based on the phenomenon of complex haploinsufficiency (CHI). Simple haploinsufficiency occurs when a heterozygous mutation in a diploid organism leads to an observable phenotype (Uhl *et al.* 2003). CHI, correspondingly, refers to the case when a strain with heterozygous mutations at two different loci has a phenotype that differs from both of the corresponding single heterozygotes (Haarer *et al.* 2007). CHI was initially referred to as unlinked non-complementation in the context of isolating *S. cerevisiae* mutants that interacted with essential genes such as tubulin (Stearns and Botstein 1988). With the advent of large-scale genetic interaction analysis methods in *S. cerevisiae*, the concept of CHI was applied to the identification of genes that interact with actin using a high throughput approach (Haarer *et al.* 2007).

Our group has previously applied CHI to *C. albicans* using transposon-based methods to generate double heterozygous mutants from a common parental or “query” strain (Bharucha *et al.* 2011). Specifically, we used transposon-mediated CHI to identify genes and pathways that interact with Cbk1 (Bharucha *et al.* 2011; Saputo *et al.* 2016), the key kinase of the Regulation of Ace2 and Morphogenesis pathway (Saputo *et al.* 2012). Recently, we reported a CHI-based genetic interaction analysis of a set of biofilm-related transcription factors (Glazier *et al.* 2017). Here, we describe the construction of a library of strains and plasmids for the systematic analysis of haploinsufficiency and CHI associated with *C. albicans* transcription factors (TF). Although CRISPR/Cas9-based approaches are likely to be very useful for genetic interaction studies (Vyas *et al.* 2015), CHI will offer advantages in a number of settings and, thus, should represent be a complementary approach to the analysis of multi-loci homozygous deletion mutants.

For example, there are at least two settings in which CHI will likely be the preferred approach. First, essential genes are not amenable to homozygous deletion analysis. Second, and more generally, genetic interactions can only be observed if the phenotypes of the single mutants are sufficiently mild to allow the detection of additional changes from that baseline. If a homozygous deletion mutant of interest completely abolishes a given cellular function, then creating a double homozygous deletion mutant with a candidate interacting gene will be completely uninformative because no additional changes in phenotype is possible. This is a reasonably frequent situation for some of the most intensely studied processes in *C. albicans* pathobiology. Homozygous TF deletion mutants that affect biofilm formation (Nobile *et al.* 2012) and hyphal morphogenesis (Homann *et al.* 2009) frequently show almost complete loss of function. For example, strains lacking Brg1 (Cleary *et al.* 2012) or Tec1 (Schweizer *et al.* 2000) are almost exclusively yeast-form under most filament inducing conditions. As such, it would be difficult to determine whether these two genes interact functionally by studying the double homozygous deletion mutants. As described herein, we have used CHI to show that these two genes, in fact, do genetically interact. A similar situation is encountered in the initial adhesion step of biofilm formation where strains lacking *BCR1* adhere at levels barely above background (Fox *et al.* 2015). Finally, *in vivo* studies of *C. albicans* homozygous deletion mutants have identified numerous “avirulent” strains; double homozygous mutants, again, would not be informative in this setting because there is little dynamic range available for measuring additional virulence variation in the double mutants.

We have constructed and characterized the phenotypes of a set of heterozygous TF deletion mutants using *in vitro* assays and an *in vivo* model of systemic candidiasis in mice. We also used a set of double heterozygous biofilm-related TF deletion strains (Rob1, Efg1, Ndt80, Bcr1, Brg1, and Tec1) derived from this library to characterize the initial adhesion and filamentation steps in biofilm formation. These genetic interaction studies indicate that Tec1 plays a previously unrecognized role in adhesion to abiotic surfaces. In addition, we have found that the set of TF genetic interactions vary significantly depending on whether the cells are adhered to abiotic surfaces or are in planktonic conditions. Under the latter conditions, we provide evidence that Rob1 plays a role in the initiation of hyphae formation based on its genetic interaction with other TFs known to function during this stage.

## Materials and Methods

### Strains and plasmids

All heterozygous and double heterozygous deletion strains of *Candida albicans* were constructed from SN152 using auxotrophic markers of *LEU2* or *HIS1* which were flanked with homologous regions of 100-500bp for targeted integration (Noble and Johnson 2005). Deletion cassettes were amplified by PCR using template DNA isolated from *C. albicans* strains in Homann’s TF deletion collection (Homann *et al.* 2009). The primers contain SbfI restriction sites, to allow release of the cassette from the plasmids. Sequences of primers used to generate deletion cassettes and to check integration are provided in Table S1. The primers amplified both the *LEU2* and *HIS1* deletion cassettes. Amplicons were cloned into pCR4TOPO (Invitrogen). To differentiate between the two auxotrophic genes, DNA was isolated from *E. coli*-transformants and digested with *Bgl*II, a restriction enzyme that cuts in the *HIS1* gene but not the *LEU2* gene. *LEU2*-containing plasmids were then sequenced to confirm the identity of the inserts.

To generate heterozygous *LEU2*-marked deletion mutants, *LEU2*-marked cassettes were excised from the plasmid by digestion with SbfI and transformed into *C. albicans* SN152 using standard lithium-acetate protocols (Hernday *et al.* 2010). Leu2+ transformants were screened for integration at the correct genomic locus by PCR using primers internal to the deletion cassette and upstream of the integration site (See Table S1 for check primers). The heterozygous nature of six strains was confirmed by quantitative PCR comparing the deletion mutant to the parental strain and the expected reduction in gene copy number was observed for each strain (Glazier *et al.* 2017); we have not performed this for each strain. Two independent isolates of each heterozygous deletion stain were archived in the collection.

The deletion cassettes contain the barcodes originally created by Homann *et al.* (Homann *et al.* 2009) and the barcode for each strain is provided in Table S1. The amplification efficiency of each barcode was verified and, in some instances, altered for poor performing barcodes. A reference strain containing a *NAT1*-marked cassette with a unique barcode was constructed by cloning the barcode sequence into pDUP3 by gap-repair recombinational cloning in *S. cerevisiae*. The resulting plasmid was integrated to an empty region of the genome as described (Gerami-Nejad *et al.* 2013).

The strains containing the *pTDH1-TEC1* allele have been described previously (Glazier *et al.* 2017). The libraries will be deposited at the Fungal Genetics Stock Center. Prior to deposit, all strains and plasmids are available upon request from the corresponding author.

### Phenotypic analysis of heterozygous deletion strains

*Candida albicans* strains were grown overnight in liquid YPD at 30° with shaking, adjusted to OD_600_ of 1.0 in PBS, and five 10-fold serial dilutions generated. The strains were then spotted onto agar plates with the appropriate media. Under a given condition, the phenotype of each heterozygous deletion strain was compared to the other heterozygous deletion strains in the pool. Strains with altered phenotypes on the initial screening plates were confirmed by repeat testing with a second independent isolate and compared to both wild type and the homozygous deletion mutant. Conditions and media for phenotyping were based on those described (Homann *et al.* 2009): YPD 30°; Spider medium; YPD hypoxia (BD GasPak EZ) 30°; 14mM CuSO_4_ in YPD; 100µM fluconazole in YPD; 15mM caffeine in YPD; YP-glycerol (30%); YNB-glycerol (30%); 1.5M NaCl in YPD and 1.5M sorbitol in YPD.

### Pooled competitive *in vitro* and *in vivo* competitive fitness assays

The Insitutional Animal Care and Use Committee at the University of Rochester approved all protocols used in this study. Mice were obtained from Charles River Laboratories. Three pools of 42-44 strains were prepared containing stains with unique barcodes by combining individual strain cultures at equal cell density. The initial inoculum pool was immediately flash frozen and DNA isolated. The pooled cultures were used to inoculate YPD cultures which were grown at 37° to mid-log phase (OD_600_ ∼1) over a time period that corresponded to ∼20 generation. The cultures were harvested and DNA was isolated. For *in vivo* experiments, the pooled cultures were used to infect 5 CD1 (ICR) mice for each pool at 5 × 10^5^ CFU/mL by tail vein injection. The mice were sacraficed 24 hr after inoculation and the kidneys were harvested. Homogenates of the kidneys were plated on YPD+gentamicin+vancomycin plates and incubated for 24 hr at 30°. Plates with ∼500 colonies were scraped of cells and DNA was isolateed. The DNA from the inoculum and from samples recovered after *in vitro* or *in vivo* growth was analyzed by quantitative PCR using primers specific to each barcode. The abundance of each strain in the recovered (R) samples was normalized to the inoculum (I) using the procedure described (Noble *et al.* 2010). The experiments were performed twice and the mean abundances for each strain were calculated. The statistical significance of deviations in R/I from the pool mean for each strain were evaluated by Student’s *t*-test using the Benjimani-Hochberg correction for multiple comparisons; statistical significance defined as *P* < 0.05.

### Two-strain competitive fitness assays

The relative, competive fitness between the reference strain and single or double heterozygotes was determined by preparing an inoculum with equal amounts of the reference strain SN250 containing a barcode integrated in a region of the genome without any ORFs and the mutant strain as determined by hemacytometry. A portion of the inoculum was flash frozen and DNA was isolated from the cells. The inoculum was used to infect mice (N= 5 for each competition) as described above for the pooled assays. The ratio of mutant/reference was determined by quantiative PCR with primers to the barcodes of the deletion mutant and reference strain, respectively.

### Adherence Under biofilm forming conditions

*C. albicans* strains were grown overnight at 30° with shaking in YPD liquid cultures. Cells were washed three times in PBS and re-suspended to an optical density (OD_600_) of 0.5 in biofilm-inducing media YET-1% sucrose (Glazier *et al.* 2017). The suspension of cells (200 µL) of cells was incubated for 90 min at 37° in 96-well, tissue culture treated, polystyrene plates. After 90 min, the media was removed and non-adherent cells were removed by careful washing with PBS. The density of adhered cells was measured by reading the optical density at 600 nM (Fox *et al.* 2015; Lohse *et al.* 2017) using a SpectraMax plate reader. At least 3 replicate wells were analyzed for each strain. The density for each mutant strain was normalized to the reference strain and referred to as normalized adherence fitness (AF). Epistasis between two mutants (X and Y) was defined as *Ɛ* = AF_XY_—AF_X_AF_Y_ where AF_XY_ is the AF of the double mutant and AF_X_ and AF_Y_ are the normalized biofilm densities of the two heterozygotes. AF_X_AF_Y_ represents the expected AF for the double mutant if there was no genetic interaction. Therefore, *Ɛ* = 0 indicate that the two mutations function independently. Double mutants whose SEM range was completely outside of the standard relative error of the expected values were defined as having an epistatic interaction (Glazier *et al.* 2017). *Ɛ* > 1 indicates a buffering interaction and *Ɛ* < 1 indicates a cooperative interaction.

### Measurement of adherent cell filament length

*C. albicans* strains were grown overnight at 30° with shaking in YPD liquid cultures. Cells were washed three times in PBS then re-suspended to an optical density (OD_600_) of 0.5 in biofilm-inducing media YET-1% sucrose (Glazier *et al.* 2017). The cell suspensions were used to inoculate chamber slides and placed at 37° for 145 min. The medium and suspended cells were removed and the slides washed with PBS to remove non-adhered cells. The slides were heated at 100° to fix cells. The adhered cells were imaged using bright-field settings on a Nikon i80 microscope with a Photometrics CoolSnap HQ_2_ camera. The length of filamented cells was determined by measuring the path-length of a line drawn along the long axis of the cell beginning at the bud neck using the NIS-Elements software package (Nikon). At least 100 cells were measure for each strain and two independent experiments were performed. The mean length was calculated and the statistical significance of the differences between strains were evaluated using the Mann-Whitney *U*-test (*P* < 0.05 was defined as statistical significance). Genetic interactions were calculated as described above after the length of each mutant was normalized to the reference strain length.

### Quantitative assays of fungal yeast/hyphae morphology using ImageStream^X^ MKII

Frozen fungal stocks were steaked onto yeast extract-peptone-dextrose (YPD) agar plates and cells were allowed to grow overnight at 30°. Yeasts from the plates were then inoculated into flasks containing 25 ml of YPD broth and incubated for 16 h at 30° with shaking at 180 rpm. Under these conditions, all *C. albicans* strains grew as budding yeasts. Cultured yeasts were washed twice with phosphate-buffered saline (PBS) and counted using a hemocytometer. Cell density was adjusted to a final concentration of 2 × 10^7^/ml with RPMI 1640 medium containing l-glutamine, but without phenol red (Gibco). Cells were seeded at 2 × 10^6^ (100 µl) per well in a 96-well round bottom plate and incubated for 6 hr at 37°. Cells were washed twice with PBS followed by incubation with a solution containing 2.5 µg/ml FM 4-64FX membrane staining dye (Thermo-Fisher Scientific Cat.# F34653) and 0.4 µg/ml calcofluor white M2R (CFW; Sigma Cat# 6258) in a modified phosphate buffer saline, pH 8.0 for 30 min at 37°. Following staining, wells were washed and resuspended in 30 µl 2% paraformaldehyde solution. Samples were analyzed with an ImageStream^X^ MKII (Millipore) image flow cytometer with a 7‐µm core at low flow rate and high sensitivity using INSPIRE software. Cells were analyzed along with 7% Speed Beads (1 μm polystyrene beads) for calibration of the flow and focus of the flow cytometer. Fluorochromes were excited with 405, 561 and 785 nm lasers and light emitted by the fluorescently‐labeled cells was collected through a ×60 objective, providing a pixel size of 0.3 μm^2^. Of the twelve channels available, channels 1 and 9 were used for bright-field images, channel 6 for side scatter light intensity and channels 5 and 7 for fluorochrome intensities of FM 4-64FX and CFW, respectively. Single‐color reference samples for each fluorochrome were generated by inclusion of cells that had been incubated with each fluorochromes separately. Images were analyzed using IDEAS software version 6.2 (Millipore). A compensation matrix was built with the data from single‐color reference samples to allow removal of spectral overlap to adjacent channels from each detection channel. Single- cell images were first gated on the focused cells with a size (area) range between 50-200 pixels (15-60 μm^2^). A dot plot of bright field area *vs.* aspect ratio (minor axes/major axes) was used to distinguish yeast from elongated hyphae population that had an aspect ratio less than 0.7 and area greater than 21 μm^2^. The percentages of yeast and hyphae populations were measured and presented as means ± standard errors.

### Data availability

The authors state that all data necessary for confirming the conclusions presented in the article are represented fully within the article.

## Results

### Construction of a library of *C. albicans* heterozygous transcription factor deletion strains and a collection of plasmid-borne transcription factor deletion cassettes

**Figure 1:**
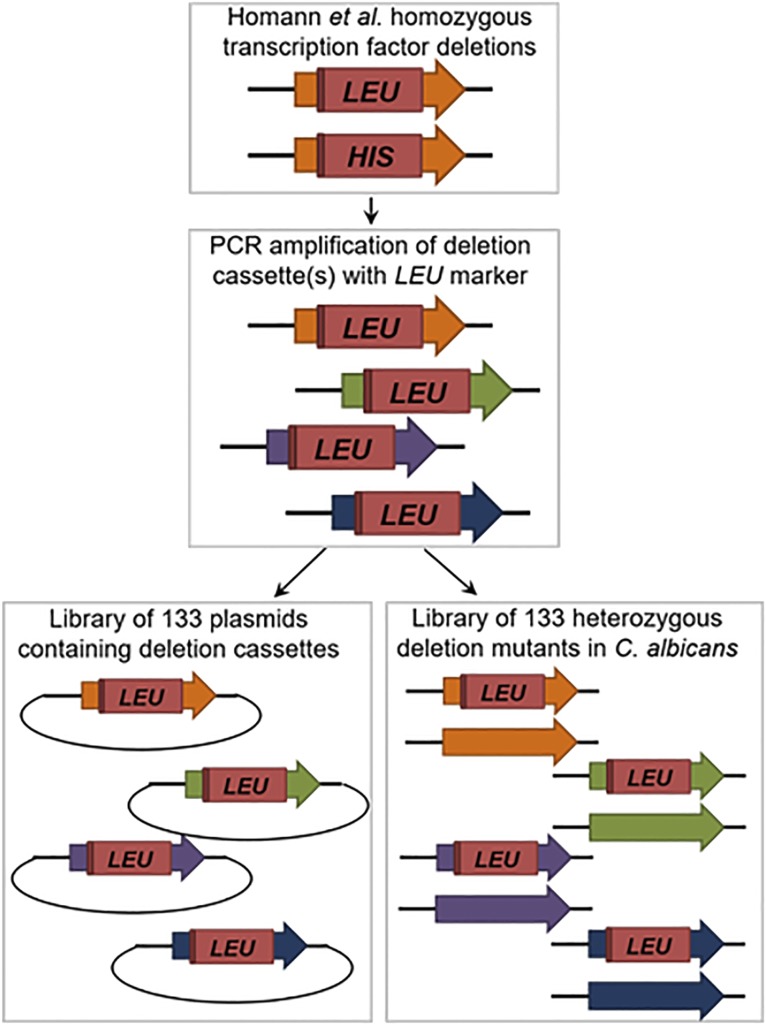
Schematic for construction of heterozygous transcription factor deletion library and *LEU2*-marked deletion cassettes.

To characterize the function of TFs regulating *C. albicans* gene expression, Homann *et al.* created a deletion library of transcription factor (TF) null mutants (Homann *et al.* 2009). In order to facilitate the application of CHI to the analysis of *C. albicans* TF networks, we generated an analogous library of 133 heterozygous deletion mutants as well as a collection of the respective deletion cassettes cloned into *E. coli* plasmids. To do so, DNA was isolated from the strains in the Homann collection (Homann *et al.* 2009) and used as templates for the PCR amplification of each TF deletion cassette. The cassettes containing the *LEU2* auxotrophic marker (Figure 1) were cloned into a TOPO-based *E. coli* vector by TA cloning techniques. The primers (Table S1) contain SbfI restriction sites to facilitate release of the cassettes. The deletion cassettes have 100-500 bp sequences homologous to the 5′ and 3′ UTR regions flanking the TF loci. The cassettes also contain barcoded signature tags adjacent to the auxotrophic markers. Each plasmid was sequenced to verify the barcodes and regions of TF-specific homology.

To construct heterozygous deletion strains (Figure 1), plasmids were digested with SbfI to release the cassettes which, in turn, were used to transform *C. albicans* strain SN152 (Leu^-^/His^-^/Arg^-^). Two independently derived strains were isolated for each TF heterozygote and the integration sites were confirmed by standard PCR analysis. Thus, we have constructed two libraries: one composed of TF *LEU2*-marked deletion cassettes in *E. coli*, and one composed of single heterozygous TF deletion mutants in *C. albicans*. The deletion collection can serve as a resource from which one can quickly generate systematic sets of double heterozygotes in a convenient *C. albicans* strain background that has been validated for *in vivo* studies (Noble and Johnson 2005). Alternatively, one could start from a query heterozygous deletion strain and generate double mutants using the set of deletion cassettes. Both of these resources will be deposited at the Fungal Genetics Stock Center.

### Phenotypic characterization of the heterozygous deletion collection

Four large-scale haploinsufficiency-based screens have been performed in *C. albicans*, previously (Uhl *et al.* 2003; Xu *et al.* 2007; Oh *et al.* 2010; Chaillot *et al.* 2017). Two were based on transposon-generated collections (Uhl *et al.* 2003; Oh *et al.* 2010) while the other two were based on a collection of heterozygous deletion mutants. Our heterozygous transcription factor deletion library corresponds to the previously characterized homozygous transcription factor deletion library and the strains have two remaining auxotrophic markers to facilitate generation of double heterozygotes as well as complementation (Homann *et al.* 2009). To characterize the collection of TF heterozygotes, we first performed a set of phenotyping experiments similar to those previously reported by Homann *et al.* for the homozygous TF deletion collection (Homann *et al.* 2009). This data provides a baseline for future CHI analyses of similar phenotypes and represents a systematic examination of the frequency of haploinsufficiency within *C. albicans* transcription factors.

**Figure 2:**
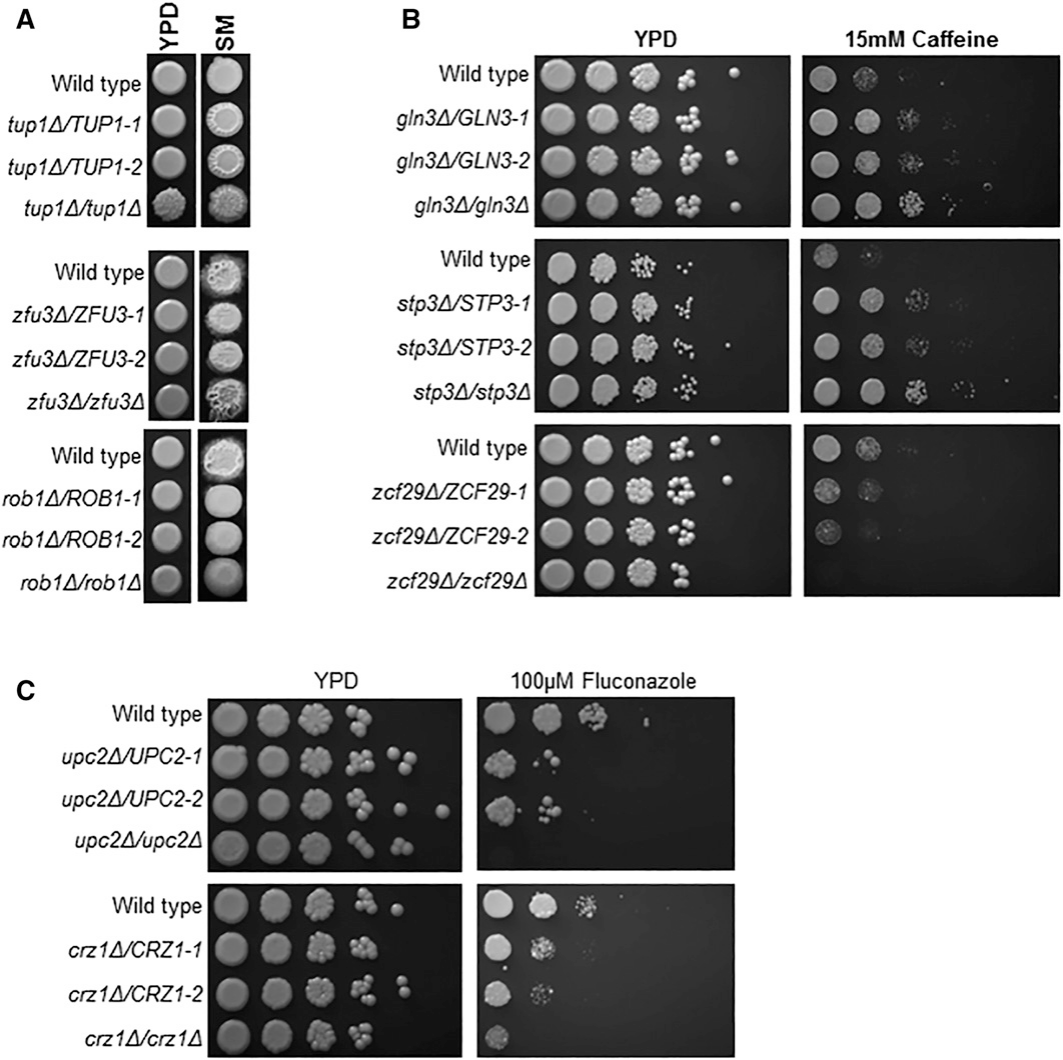
Representative haploinsufficent and haploproficient strains. Ten-fold dilution series of the indicated strains were spotted on agar plates and incubated at either 37°C (A) or 30°C for 1-3 days and photographed. Two independent isolates of each heterozygous strain were plated as noted by -1 and -2. A. The indicated strains were plated on YPD (yeast peptone dextrose) and SM (Spider medium). B. The strains were plated on YPD and YPD+15 mM caffeine. C. The indicated strains were plated on YPD+1%DMSO and YPD+1%DMSO+fluconazole.

We examined the growth of 133 of the 137 strains under the following conditions: YPD 30°, Spider agar, YPD hypoxia, YPD 14mM copper, YPD 100µM fluconazole, YPD 15mM caffeine, YP-Glycerol, YNB-Glycerol, YPD 1.5M NaCl and YPD 1.5M sorbitol. These conditions were not as extensive as that performed for the homozygous deletion set but covered a range of biologically distinct physiological states and stresses (Homann *et al.* 2009). The results of this phenotyping are summarized in Table 1. For the majority of conditions, we observed no evidence of haploinsufficiency or haploproficiency. Filamentation was the phenotype for which we observed the highest rate of haploinsufficiency/haploproficiency: 13/133 (10% of mutants). Representative examples of haploproficient (*TUP1*, orf19.6109) and haploinsufficient (*ROB1*, orf19.4998; *ZFU3*, orf19.6888) strains are shown in Figure 2A. For all but two of the mutants (*ZFU3*, orf19.6888) and *NDT80*, orf19.2119), the filamentation phenotypes were concordant with the corresponding homozygous deletion mutant. Vandeputte *et al.* have reported that the homozygous deletion of *ZFU3* (orf19.6888) is hyperfilamentous (Vandeputte *et al.* 2011). The homozygous deletion of *NDT80* (orf19.2119), on the other hand, is deficient in filamentation. As we have recently reported, the increased filamentation shown by *ndt80*Δ (orf19.2119) is dependent on Rob1 (orf19.4998) and Tec1 (orf19.5908) (Glazier *et al.* 2017). Although we have not investigated the mechanism of this observation, it seems that loss of one allele may trigger a dysregulated compensatory process that is dependent on the remaining allele. When both alleles are missing, the compensatory process is not able to support filamentation.

**Table 1:** Heterozygous TF mutants with *in vitro* phenotypes

**Phenotype**	**Heterozygous Transcription Factor Mutant^1^**
**Filamentation (Spider Medium)**	*TUP1*, *NRG1*, *WOR1*, *RIM101*, *SFL1*, *CUP9*, *NDT80*, ***ROB1*, *BRG1***, ***EFG1***, *STP2*, *AAF1*, *ZFU3*
**Caffeine**	***GLN3***, ***STP3***, *ZCF29*
**Fluconazole**	***UPC2*, *CRZ1***
**Filamentation (Hypoxia)**	*AHR1*, *STP4*

1 **Bold** denotes mutants with haploinsufficient phenotype; standard text indicates mutants with haploproficient.

Two heterozygotes, *upc2*Δ and *crz1*Δ, were hypersensitive to fluconazole, which is consistent with their well-characterized roles in the cellular response to this drug (Vasicek *et al.* 2014; Onyewu *et al.* 2004). Upc2 (orf19.391) is a key regulator of ergosterol biosynthesis genes while Crz1 (orf19.7359) is an important transcriptional regulator under control of the calmodulin-calcineurin axis. Two heterozygotes (*GLN3*, orf19.3912 and *STP3*, orf19.5915) were resistant to caffeine (Figure 2B), a drug that interacts with the TOR signaling pathway (Reinke *et al.* 2006), while a third (*ZCF29*, orf19.5133) was more sensitive. The caffeine phenotypes for all three heterozygotes are concordant with their corresponding homozygous deletion mutants (Homann *et al.* 2009). The TOR pathway is critically involved in the regulation of amino acid and nitrogen metabolism and, consistent with that function, *GLN3* (Dabas and Morschhauser 2007) and *STP3* (Martinez and Ljungdahl 2005) are also involved in nitrogen metabolism. *ZCF29* (orf19.5133) has recently been shown to be important in drug resistance (Shekhar-Guturja *et al.* 2016). Homozygous deletions of *GLN3* (orf19.3912) *STP3* (orf19.5915) and *ZCF29* (orf19.5133) also show altered susceptibility to rapamycin (Homann *et al.* 2009).

### Low rate of haploinsufficiency in rich media During pooled competitive growth

The heterozygous TF mutants each contain a barcode and, therefore, are amenable to signature tag methods to quantitate phenotypic differences in pooled samples (Noble *et al.* 2010). We, therefore, asked if any of the TF heterozygotes showed growth defects in rich medium (YPD) at 30°. Pools containing 44-48 mutants were prepared by combining equal numbers of cells. The DNA from a sample of the resulting pool was isolated as the input and the ratio of strains was determined by quantitative PCR using barcode specific primers. The pool was then used to inoculate an YPD culture which was incubated for 20 generations to a final cell density between 0.5-1.0 OD_600_. The cells were harvested, the DNA was isolated, and the abundance of each strain was determined by quantitative PCR. The ratio of each strain in the recovered sample relative to the input sample was determined using the method described by Noble *et al.* and is described in more detail in the Materials and Methods (Noble *et al.* 2010).

We defined a significant change in the abundance of a heterozygote strain as a statistically significant (*P* < 0.0001, Student’s *t*-test with the Benjamani-Hochberg correction for multiple comparisons) twofold increase or decrease (± 1 log_2_) in abundance relative to the inoculum at 24 hr. Although *uga33*Δ showed increased fitness, none of the heterozygous deletion mutants showed a competitive disadvantage under these conditions (see Table S2 for data). These observations are consistent with our plate-based assays in which we saw no apparent growth defect in rich media. The rate of haploinsufficiency in the *S. cerevisiae* genome has been estimated to be approximately 3% (Deutschbauer *et al.* 2005). Thus, if these were a random set of mutants, then we would expect 3-4 haploinsufficient strains at most. As such, it is not surprising that we did not observe growth defects. This set of data does, however, serve to validate our primers and barcodes and provides a benchmark for future studies using double heterozygous mutants or heterozygous mutants under other conditions.

### Pooled competitive growth assay in a murine model of disseminated candidiasis identifies haploinsufficient transcription factor mutants

Previous studies of TF homozygous deletion mutants using signature-tagged approaches in murine models of disseminated candidasis have identified a sub-set that is required for infection (Amorim-Vaz *et al.* 2015). We, therefore, carried out a similar experiment using pools of heterozygous TF mutants and the signature-tagged approach described above. The pools of mutants were used to infect mice by tail vein injection. The mice were killed at 24hr and 48hr, the kidneys were harvested, and the homogenates plated on YPD plates. After 24hr at 30°, the plated cells were collected and the DNA was isolated. The relative proportions of the pooled strains were determined by quantitative PCR as described for the *in vitro* experiment. We identified heterozygotes that a showed statistically significant (*P* < 0.0001, Student’s *t*-test with the Benjamani-Hochberg correction for multiple comparisons), twofold or more increase or decrease (± 1 log_2_) in abundance relative to the inoculum. Twelve heterozygous mutants met these criteria; eight showed a competitive fitness disadvantage while four were more fit. The genes with altered competitive fitness and their annotations are listed in Table 2; the full set of data for the pools are provided in Table S3.

**Table 2:** HeterozygousTF mutants with altered infectivity in murine model of disseminated candidiasis

**Transcription Factor**	**Function^1^**	**Log_2_(R/I)^2^ (24hr, 48hr)^3^**
***ACE2*** (orf19.6124)	Daughter specific, constitutive pseudohyphae, adherence	**-2.1,** 0.36
***CRZ2*** (orf19.2356)	Related to calcineurin regulated Crz1, adherence	0.95, **3.7**
***FGR15*** (orf19.2054)	Involved in filamentous growth, caspofungin-induced	-**1.5**, -2.2
***RME1*** (orf19.4438)	Similar to meiotic regulator in *S. cerevisiae*, White cell-associated	**-2.3,** -3.0
***RFX1*** (orf19.3865)	Represses filament specific gene expression, hyper-filamentous	−0.9, **-4.1**
***SFL1*** (orf19.454)	Heat shock-like regulator of morphogenesis, hyper-filamentous	**-2.1, -2.7**
***SFL2*** (orf19.3969)	Heat shock-like regulator of morphogenesis, hypo-filamentous	**-4.8, -7.8**
***SUC1*** (orf19.7319)	Regulates glucosidase expression, adherence	**-3.9, -4.0**
***TRY6*** (orf19.6824)	Adherence	**1.7, 4.0**
***UGA33*** (orf19.7317)	Similar to regulator of GABA metabolism, adherence	**3.0,** 0.9
***ZCF25*** (orf19.4568)	Uncharacterized	**-1.4,** -0.24
***ZCF26*** (orf19.4573)	Uncharacterized	**1.3,** 0.9

1 Functional annotations are based on entries in the Candida Genome Database (http://www.candidagenome.org)

2 R indicates recovered from animal and I indicates inoculum. See materials and methods for full details of calculation.

3 Bold denotes statistically significant Student’s *t* test with Benjamini-Hochberg correction for multiple comparisons.

Five of the twelve mutants (*ace2Δ* orf19.6124, *suc1Δ* orf19.7319, *tyr6Δ* orf19.6824, *crz2Δ* orf19.2356, and *uga33Δ* orf19.7317) were identified by Finkel *et al.* as being required for adherence *in vitro* (Finkel *et al.* 2012). The experiment examined two time points early in the infection process (24 hr and 48 hr), times when differences in endothelial attachment may be quite prominent. Therefore, it seems reasonable that strains with altered adherence would be identified in this experiment. Of these five mutants, *uga33*Δ (orf19.7317) and *tyr6*Δ (orf19.6824) showed increased fitness while the other three strains showed reduced fitness. The *uga33*Δ (orf19.7317) also showed increased fitness during growth in rich medium. It is, therefore, possible that this mutant has a competitive growth advantage *in vivo* as well. Although we cannot exclude a proliferation defect or delay for the other mutants, the lack of haploinsufficiency for these strains during growth in rich media reduces the likelihood that general growth defects contribute to the decreased infection fitness. We selected mutants with roles in *in vitro* adherence (*ace2Δ*, orf19.6124 and *suc1Δ*, orf19.7319) and morphogenesis (*sfl1Δ*, orf19.454 and *sfl2Δ*, orf19.3969) to confirm the pooled results with competitive experiments with wild type strains.

Ace2 (orf19.6124), the TF regulated by the RAM signaling pathway (Saptuo *et al.* 2015), and Suc1 (orf19.7319), a zinc finger TF originally identified as a regulator of sucrose utilization (Kelly and Kwon-Chung 1992), both were identified by Finkel *et al.* as regulators of adherence (Finkel *et al.* 2012). The homozygous deletion mutant of *ACE2* (orf19.6124) also has been shown to have a virulence defect in disseminated candidiasis (Kelly *et al.* 2004). A previous screen of pooled homozygous TF deletion mutants without morphology defects found that *suc1*ΔΔ (orf19.7319) has a competitive fitness defect when animals are harvested late in infection (Pérez *et al.* 2013). Finally, we also tested the haploinsufficiency of *sfl1*Δ and *sfl2*Δ, two heat shock-type TFs with opposite effects on filamentaton (Znaidi *et al.* 2013). The homozygous deletion mutant of *SFL1* (orf19.454) shows increased filamentation while *sfl2Δ* (orf19.3969) has a filamentation defect. As noted above, *slf1*Δ is hyperfilamentous while *sfl2*Δ was similar to wild type. In addition, *SFL1* (orf19.454) is required for virulence in a disseminated candidiasis model (Li *et al.* 2007) while *SFL2* (orf19.3969) was dispensable (Spiering *et al.* 2010).

**Figure 3:**
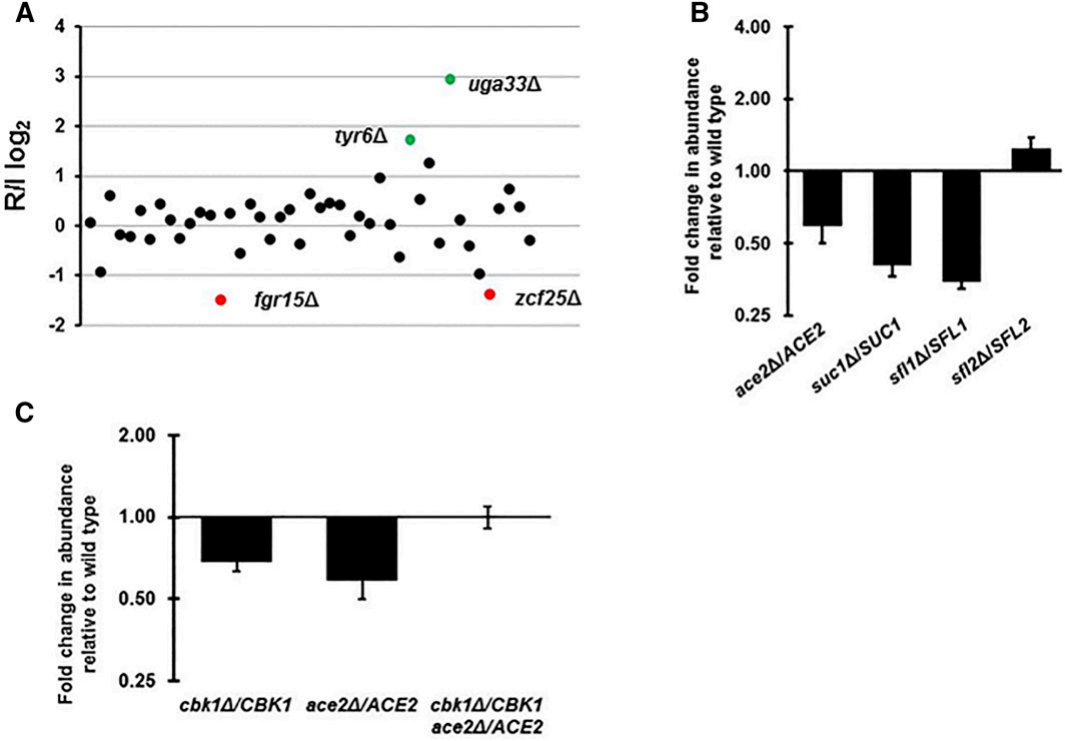
TF heterozygous strains have altered competitive fitness at early stages of dissemination in a murine model of candidiasis. A. CDR1 mice were infected with pools of orthogonally barcoded heterozygous deletion mutants and harvested at 24 or 48 hr post infection (N = 5 for each time point). The competitive fitness was calculated as described in Materials and Methods. Competitive fitness data from representative pool of 48 heterozygous strains is shown. Mutants with statistically significant (*P* < 0.05, Student’s *t*-test with Benjamani-Hochberg correction for multiple comparisions) decreased (red points) or increased (green points) competitive fitness relative to the median pool R/I. B and C. Competitive fitness of the indicated strain relative to a wild type reference strain. Bars indicate means of 3-5 independent mice with error bars indicate standard error of the means.

As shown in Figure 3B, three (*ACE2* orf19.6124, *SUC1* orf19.7319, and *SFL1* orf19.454) of the four heterozygous strains showed significantly reduced fitness relative to the wild type reference strain in head to head competitions at 24 hr. The *sfl2*Δ (orf19.3969) showed slightly increased fitness but this is not likely to be biologically significant. These data are consistent with the findings of Spiering *et al.* which showed that *SFL2* (orf19.3969) was not required for infectivity at 3 days (Spiering *et al.* 2010). Therefore, our pooled screening with 48 strains had a true positive rate of 75%; it is likely that the specificity of the screening experiment could be increased with the use of smaller pools.

Two of the genes that we chose to validate, *ACE2* (orf19.6124) and *SFL1* (orf19.454) are constitutively filamentous as homozygous deletions (Kelly *et al.* 2004; Saputo *et al.* 2012; Znaidi *et al.* 2013). This morphological phenotype complicates infectivity and virulence studies using tail vein infections. The *ace2*Δ (orf19.6124) mutant is not hyperfilamentous *in vitro*, nor did it have a growth phenotype. Therefore, our data indicate that Ace2 (orf19.6124) may have effects on infectivity that are independent of its role in the regulation of morphology. Since the *sfl1*Δ (orf19.454) mutant is hyperfilamentous, it remains possible and, indeed likely, that its apparent infectivity defect is due to this morphological feature.

Ace2 (orf19.6124) is regulated by the RAM network through phosphorylation by Cbk1 (orf19.4909) (Saputo *et al.* 2012). To our knowledge, the virulence of Cbk1 (orf19.4909) has not been assessed previously. This may be due to the fact that *cbk1*ΔΔ (orf19.4909), like *ace2*ΔΔ (orf19.6124), has a profound cell separation defect that complicates interpretation of *in vivo* studies (Saputo *et al.* 2012). Based on the phenotype showed by the *ACE2* (orf19.6124) heterozygote, we hypothesized that the *CBK1* (orf19.4909) heterozygote may also show haploinsufficiency with respect to initial infection. Therefore, we asked if *cbk1*Δ (orf19.4909) showed a competitive disadvantage relative to WT in the early fungal burden experiment. Consistent with the behavior of the *ACE2* heterozygote (orf19.6124), *cbk1*Δ/*CBK1* (orf19.4909) is out-competed by WT at 24hr (Figure 3C).

Finally, we generated the double heterozygote of *cbk1*Δ *ace2*Δ and examined its infectivity relative to each of the single heterozygotes. Surprisingly, the double mutant was restored to WT infectivity (Figure 3C). One possible explanation for this observation is that we had previously observed that deletion of one *ACE2* (orf19.6124) allele in the *cbk1*Δ (orf19.4909) background restored filamentation to the haploinsufficient parental *cbk1*Δ (orf19.4909) strain (Saputo *et al.* 2016). The homozygous *cbk1*ΔΔ (orf19.4909) strain is completely unable to form filaments while the *ace2*ΔΔ (orf19.6124) is hyper-filamentous. It is, therefore, possible that the increased filamentation displayed by the double *cbk1*Δ *ace2*Δ correlates with its improved infectivity relative to the two single heterozygotes.

### Complex haploinsufficiency-based analysis reveals role for Tec1 During initial adherence Under biofilm forming conditions

**Figure 4:**
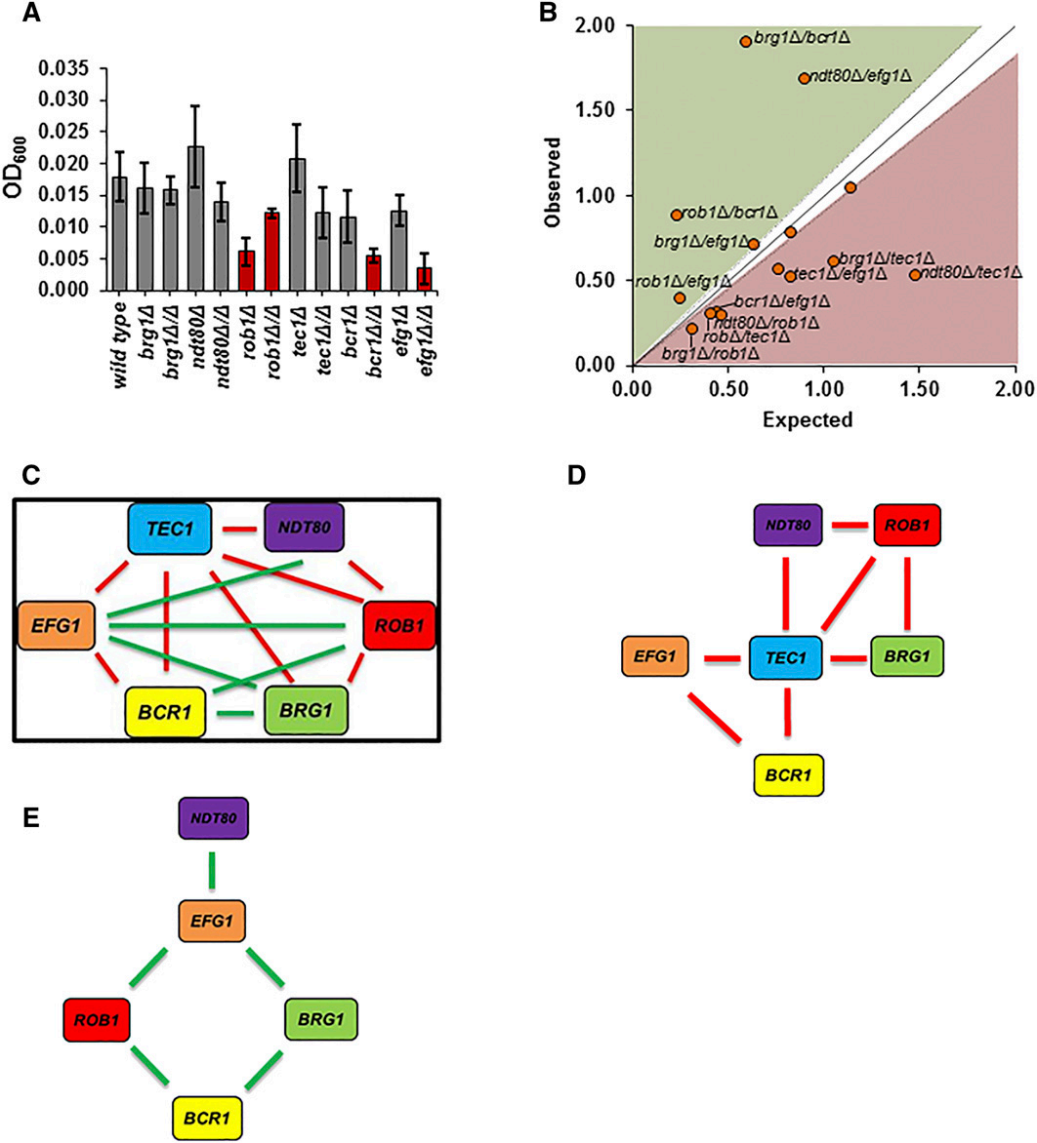
Haploinsufficiency and genetic interactions of biofilm TFs during the initial adherence stage of biofilm formation. A. The indicated strains were incubated in microtiter plates in YETS medium at 37°C for 90 min before non-adherent cells were removed by careful washing with PBS. The bars indicate the mean OD_600_ for each strain (triplicate) with error bars indicating standard deviation. Strains with a statistically significant (Student’s *t*-test *P* < 0.05) change from the reference strain are indicated by red bars. B. The expected adherence fitness (*AF* = Mutant OD_600_/Reference OD_600_) for a given double mutant (*AF*_mutA_ X *AF*_mutB_) is plotted on x-axis for each biofilm TF double heterozgote and the observed AF for the double mutant is plotted on the y-axis. Mutants that fall within the zone defined by the diagonal (ε = 0 ± estimated error) have no genetic interaction while those above the diagonal (green) display alleviating interactions while those below (red) show aggravating interactions. C. Network of biofilm TF interactions during the adhesion step. Green edges indicate alleviating interactions; red edges indicate aggravating interactions. D. Aggravating interaction network centered on Tec1. E. Alleviating interaction network.

As part of a separate study of the genetic interaction network for mature biofilm formation (Glazier *et al.* 2017), we used our library to construct a complete set of double heterozygous TF deletion mutants corresponding to the six member biofilm TF network (Efg1 (orf19.610), Bcr1 (orf19.723), Tec1 (orf19.5908), Ndt80 (orf19.2119), Rob1 (orf19.4998), and Brg1 (orf19.4056)) elucidated by Nobile *et al.* (Nobile *et al.* 2012). Recently, the Johnson group reported the effect of homozygous deletion mutants of these TFs on the initial adherence step of biofilm formation (Fox *et al.* 2015). Accordingly, we used the corresponding set of double heterozygotes to identify functionally important interactions between the TFs during initial adhesion to abiotic surfaces. We used the same optical density-based assay to evaluate initial adhesion as Fox *et al.* (Fox *et al.* 2015); however, we used YETS medium instead of Spider medium because it gave less sample-to-sample variation (Fox *et al.* 2015). As shown in Figure 4A, three homozygous deletion stains showed reduced initial adhesion (*EFG1* (orf19.610), *ROB1* (orf19.4998), and *BCR1* (orf19.723)) while only *ROB1* (orf19.4998) showed haploinsufficiency.

To characterize genetic interactions between mutants, we normalized the optical density for each mutant to wild type and will refer to this ratio as the adhesion fitness (*AF*). The multiplicative model was used to define genetic interactions (Mani *et al.* 2008); this model is the same as that used by Boone and co-workers for the genome-wide *S. cerevisiae* experiments (Constanzo *et al.* 2016). In the multiplicative model, the expected fitness of a double mutant derived from a pair of non-interacting genes is equal to the product of the fitness values for each individual mutant: *AF_12_* = *AF*_1_ x *AF*_2_ (Mani *et al.* 2008). This is also called genetic neutrality and indicates that the two genes affect the phenotype independently. If *AF*_12_ < *AF*_1_ x *AF*_2_, then the phenotype of the double mutant is more severe than predicted by the model and these genes show an aggravating interaction. If, on the other hand, *AF*_12_ > *AF*_1_ x *AF*_2_, then the two genes show an alleviating or suppressing interaction.

Traditionally, genetic interactions are expressed as Ɛ = *AF*_12_ – (*AF*_1_ x *AF*_2_) where Ɛ > 0 is an aggravating interaction, Ɛ < 0 is an alleviating interaction and Ɛ = 0 is no interaction. We have provided Ɛ values for each double mutant pair in Table S2. However, as a more easily evaluated graphical representation of the data, we plotted the expected AF *vs.* the observed AF for the all double mutants with the estimated error. Mutants that plotted above the diagonal show aggravating interactions while those below the diagonal display alleviating interactions. In addition, we required that a double mutant show a statistically significant difference from the expected value to be classified as an interacting pair of genes (Student’s *t*-test, *P* < 0.05).

An extensive set of genetic interactions was identified of the adhesion phenotype (Figure 4B) with only two gene-pairs out of the total 15 gene-pairs showing no interaction (See Table S4 for complete set of adhesion data and ε calculations). Three TFs [Tec1 (orf19.5908), Rob1 (orf19.4998), and Efg1 (orf19.610)] interacted with all five of the other TFs (Figure 4C). Interestingly, Tec1 (orf19.5908) showed an aggravating interaction with all other TFs. Thus, the aggravating interaction network for adhesion is centered on Tec1 (orf19.5908) as a central node (Figure 4D). This relationship would not have been identified using single gene analysis because the *tec1*Δ/Δ (orf19.5908) mutant does not have a defect in adhesion (Fox *et al.* 2015). Consequently, it appears that Tec1 (orf19.5908) functions in an ancillary or redundant manner to regulate the initial adhesion step of biofilm formation. In addition, our data indicate that Ndt80 (orf19.2119) and Brg1 (orf19.4056) also contribute to this step but likewise do so in a manner that overlaps with other TFs because their homozygous deletion mutants do not show a phenotype. Furthermore, our data are consistent with the notion that multiple TFs have overlapping functions during this step of biofilm formation.

**Figure 5:**
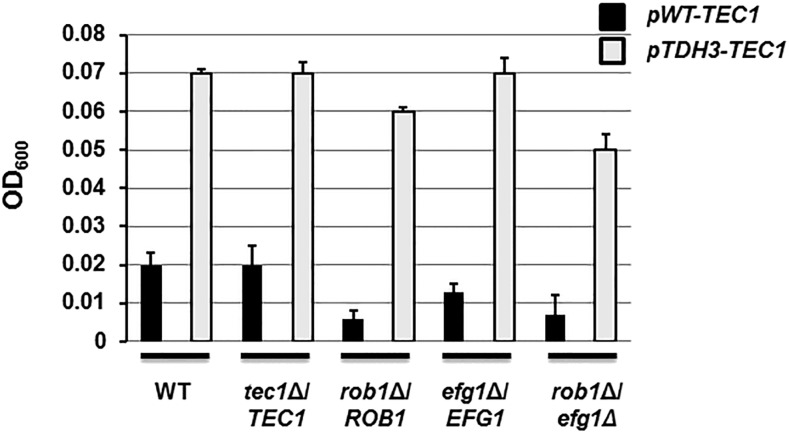
Overexpression of *TEC1* increases adhesion of WT and suppresses adhesion defects of other biofilm TF deletion mutants. The mean adhesion of the indicated strains with and without an allele of *TEC1* under the control of the strong, constitutive TDH1 promoter is shown by the bars with error bars indicating the standard deviation of duplicates or triplicates.

If *TEC1* (orf19.5908) plays a significant but ancillary role in adhesion, then its over-expression could be expected to suppress the adhesion defects displayed by other mutants. Therefore, we placed *TEC1* (orf19.5908) under control of the strong, constitutive promoter *TDH1* using the methods previously described by Finkel *et al.* (Finkel *et al.* 2012). As shown in Figure 5, over-expression of *TEC1* (orf19.5908) increases adhesion in WT and *tec1*Δ (orf19.5908) strains. Additionally, increased expression of *TEC1* (orf19.5908) in all of the adhesion-deficient strains that we tested led to increased adhesion. Taken together, these data suggest that *TEC1* (orf19.5908) plays an important but ancillary role in the initial adhesion step of biofilm formation. In this role, it would function cooperatively with other TFs but be dispensable when a full complement of its functional partners is present.

A second interaction network consisting of only alleviating or suppressive interactions also emerges from adhesion data. In this case, a four-node structure connects Efg1 (orf19.610), Brg1 (orf19.4056), Bcr1 (orf19.723), and Rob1 (orf19.4998). Ndt80 (orf19.2119) has an alleviating interaction only with Efg1 (orf19.610). Alleviating interactions between two genes can indicate that the two genes function in a linear pathway and a “diminishing returns” effect limits the severity of two mutations that affect genes in the same pathway (Mani *et al.* 2008). Based on this interpretation, each of the nodes in the alleviating network would function in parallel pathways where reductions in gene dosage along one pathway are buffered. However, reduction in two genes/nodes that are not linearly-related disrupts both parallel pathways and leads to an aggravating interaction.

### The biofilm TF network buffers adherent *C. albicans* cells From genetic perturbations that affect filament length

**Figure 6:**
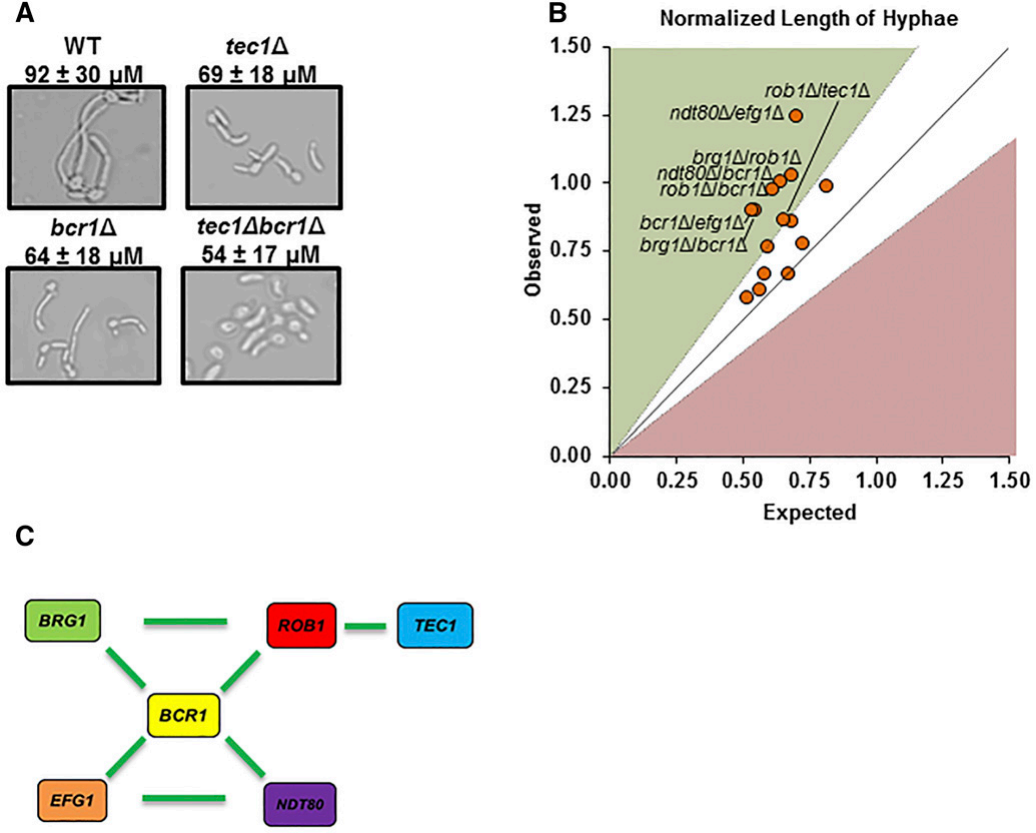
The genetic interactions of biofilm TFs during filamentation of adherent cells are distinct from adhesion and mature biofilm formation. A. Representative micrographs used to measure the filament length of the indicated strains. The lengths were determined as described in the Materials and Methods with the mean and standard deviation for the indicated strains derived from the measurement of at least one hunderd cells. B. The normalized filament length (mutant length/WT length) expected for a given double mutant was calculated as the product of the normalized mutant lengths of the two single mutants and is plotted on the y-axis. The observed filament length for a given mutant is plotted on the x-axis. Mutants that fall within the zone defined by the diagonal (ε = 0 ± estimated error) have no genetic interaction while those above the diagonal (green) display alleviating interactions while those below (red) show aggravating interactions. C. The genetic interaction network of adherent cell filamentation centered on *BCR1*. Green indicates alleviating interactions.

The second step of biofilm formation is considered to be filamentation of the adherent cells (Desai and Mitchell 2015). To our knowledge, this step of biofilm formation has not been probed genetically. To do so, we developed an assay to directly measure the length of hyphae adhered to glass chamber slides. We inoculated each chamber slide with a given strain in YETS medium. After 2 hr, the chambers were washed to remove non-adherent cells. The adherent cells were heat fixed and then examined by light microscopy. The length of the filament was measured using commercially available software. The filament was defined as the region between the angle of the mother cell-filament junction and the distal tip of the filamentous portion of the cell. We did not distinguish between pseudohyphae or hyphae. Mutants that formed no discernible hyphae were excluded from analysis. A representative set of images are shown for the reference strain, *tec1*Δ (orf19.5908), *bcr1*Δ (orf19.723), and *tec1*Δ *bcr1*Δ are shown in Figure 6A.

We first examined adherent cell filament formation using the homozygous biofilm TF deletion mutants. Consistent with previously reported filamentation phenotypes under other conditions (Homann *et al.* 2009), none homozygous deletion biofilm TF mutants were able to filament with the exception of *NDT80* (orf19.2119) which formed shorter filaments than the reference strain. The average reference strain filament length was 92 ± 30 µm (Figure 6A). Next, we determined whether the heterozygous TF deletion mutants were haploinsufficient or haploproficient. The filaments formed by five of the six heterozygous TF deletion mutants were between 11 and 20 µm shorter than the reference strain in a statistically significant manner (Mann-Whitney *U*-test, *P* < 0.05). The complete set of filament length data and calculations for ε values are provided in Table S5. Although the filaments formed by *ndt80Δ* (orf19.2119) were longer than those formed by the reference strain, the difference was not statistically different that wild type.

Next, the filament length of the double heterozygous biofilm TF mutants was measured; the genetic interactions within the double mutants were determined using calculations described earlier. We defined an interaction as a filament length that differed from the expected value by ≥ 30% based on the standard distribution of the lengths. As shown in Figure 6B, 7 out of 15 double heterozygote mutants showed genetic interactions. A number of double mutants had statistically significantly shorter filaments than either single mutants but ε = 0 for these strains, indicating that the genes did not interact and, hence, function independently. For example, the filaments of the *tec1 bcr1* double heterozygote are shorter than either single heterozygote but are equal to the length expected if each single mutant contributed independently to the observed phenotype (Figure 6A/B). All of the double mutants that show genetic interactions have filaments that are longer than predicted by the multiplicative model, indicating that only alleviating or suppressive interactions were observed.

As shown in Figure 6C, *BCR1* (orf19.723) interacts with all five of the network TFs. Consequently, *BCR1* (orf19.723) forms the central hub of a pair of interlocking three-node motifs involving *ROB1-BRG1* and *EFG1-NDT80* (Zhang *et al.* 2005). Because this network is made entirely of alleviating interactions, it is quite distinct from either network observed during initial adhesion. It appears that the interacting TFs buffer the network against genetic perturbation with *BCR1* (orf19.723) playing a key role in this effect. These observations, taken together with the adherence analysis, suggest that Tec1 (orf19.5908) and Bcr1 (orf19.723) play important but distinct roles during the initial stages of *C. albicans* interaction with abiotic surfaces under biofilm inducing conditions.

### Distinct TF interactions regulate filament length Under planktonic conditions relative to biofilm conditions

**Figure 7:**
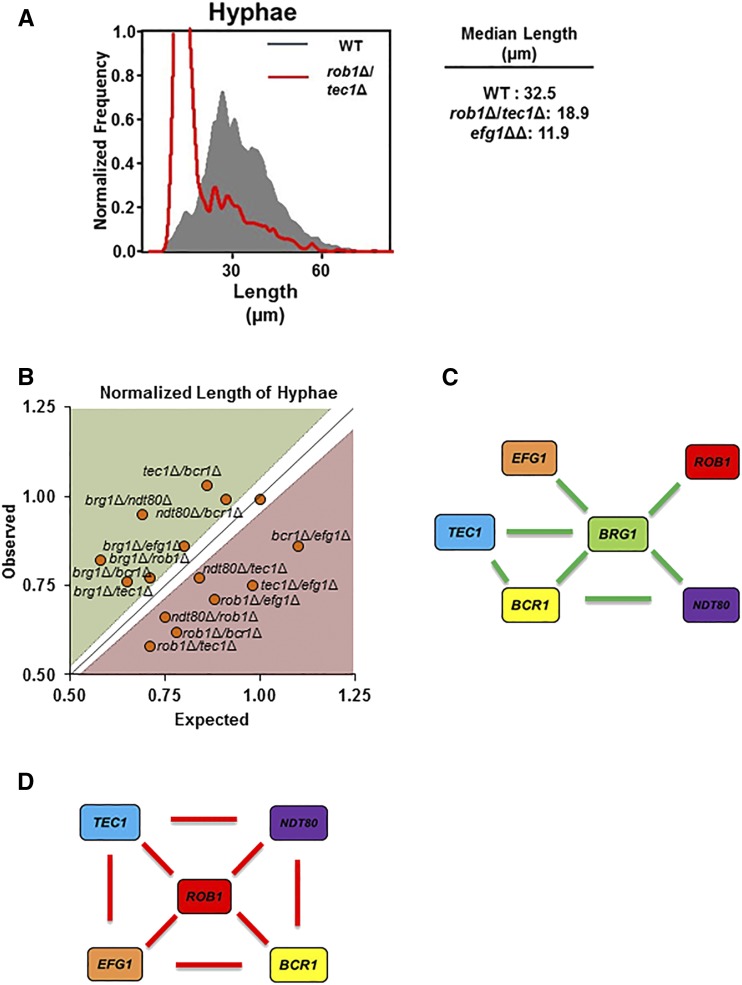
The genetic interaction network of biofilm TFs during planktonic filament formation is distinct from adherent cell filamentation. A. Plot of the distribution of filament lengths for WT and *rob1*Δ *tec1*Δ derived ImageStream-based analysis along with mean length (µm) ± SD for both strains. B. The normalized filament length (mutant length/WT length) expected for a given double mutant was calculated as the product of the normalized mutant lengths of the two single mutants and is plotted on the y-axis. The observed filament length for a given mutant is plotted on the x-axis. Mutants that fall within the zone defined by the diagonal (ε = 0 ± estimated error) have no genetic interaction while those above the diagonal (green) display alleviating interactions while those below (red) show aggravating interactions. C. Genetic interaction network for alleviating interactions centered on *BRG1*. D. Genetic interaction network for aggravating interactions centered on *ROB1*.

The extensive literature on *C. albicans* filamentation suggests that the genetic requirements for this process are dependent on specific stimuli (Saputo *et al.* 2016; Azadmanesh *et al.* 2017). We, therefore, were interested in comparing the genetic interactions of the biofilm regulators during planktonic filamentation to those for cells adhered to an abiotic surface. To do so, we took advantage of a flow cytometry-based imaging assay that both distinguishes yeast from filamentous cells and allows the measurement of filament length (Figure 7A). Among a number of advantages to this approach, it allows the rapid analysis of very large numbers of cells and, thereby, enables the detection of subtle changes in morphologic distribution with high statistical power. This is particularly advantageous for the analysis of simple and complex haploinsufficiency where phenotypes for individual mutants can be modest but informative.

The biofilm-inducing medium used in the experiments described above (YETS) is not an efficient inducer of hyphae under planktonic conditions and, therefore, we used RPMI tissue culture medium at 37° for six hours to induce hyphae. Under these conditions, 90% of the reference strain SN250 formed hyphal cells (Figure 7A) while the homozygous deletions formed no filaments. All of the heterozygous and double heterozygous deletion mutants formed filaments with measurable lengths. The data for the full set single and double heterozygotes as well as the wild type control are provided in Table S6. Two heterozygous mutants, *brg1*Δ (orf19.4056) and *rob1*Δ (orf19.4998), showed haploinsufficiency with statistically significant reductions in mean filament length of 27% and 20%, respectively.

**Figure 8:**
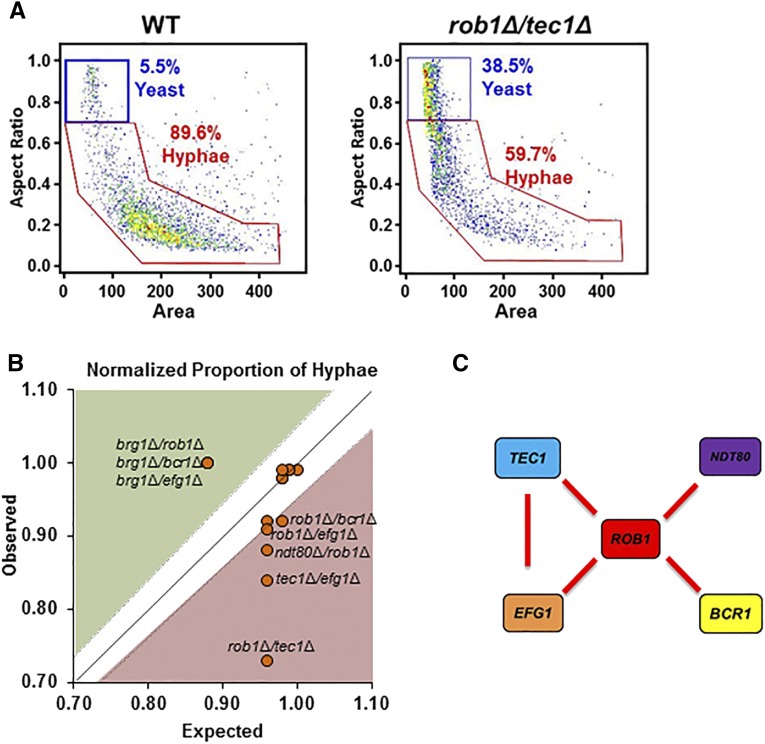
The genetic interaction network of biofilm TFs affecting the proportions of yeast and filamentous cells under planktonic conditions is similar to that for length under planktonic conditions. A. ImageStream gating diagrams showing WT and *rob1*Δ *tec1*Δ yeast and filamentous cells. B. The expected normalized proportion of filamentous cells (mutant/WT) for the double mutants was calculated as the product of the normalized filament proportion of the two single mutants and is plotted on the y-axis. The observed normalized filament proportion for a given mutant is plotted on the x-axis. Mutants that fall within the zone defined by the diagonal (ε = 0 ± estimated error) have no genetic interaction while those above the diagonal (green) display alleviating interactions while those below (red) show aggravating interactions. C. The genetic interaction network for alleviating interactions.

Using the definitions for genetic interactions described above for the analysis of cell length of adhered cells, eight aggravating and five alleviating interactions were identified. The *brg1*Δ (orf19.4056) mutant only participated in alleviating interactions and is a hub connecting a network of alleviating interactions as shown in Figure 8B. Indeed, deletion of any other TF in the *brg1*Δ background suppressed its defect in filament length. In addition, the *BRG1* (orf19.4056)-centered network contains two three-TF modules within the alleviating interaction network. This suggests that reduced copy number in other TFs triggers a compensatory response that overcomes the defects associated with reduced *BRG1* (orf19.4056) copy number.

In contrast to the filamention of the adherent cells, five aggravating interactions were observed under planktonic conditions. Specifically, the set of interactions organizes into a highly connected network centered on *ROB1* (orf19.4998). *ROB1* (orf19.4998) interacts with all of the other TFs with the exception of *BRG1* (orf19.4056) in a cooperative manner. In addition, each of the *ROB1* (orf19.4998) interacting genes also interacts cooperatively with two other TFs in this network. Thus, Rob1 (orf19.4998) and Brg1 (orf19.4056) have very distinct functional interactions during planktonic filamentation formation. Rob1 (orf19.4998) functions cooperatively with multiple TFs during planktonic filamentation. Brg1 (orf19.4056), on the other hand, does not appear to cooperate with any of the other TFs. However, reduced gene dosage of the TFs that function in the Rob1 (orf19.4998) network appears to lead to a compensatory response that suppresses the effect of decreased Brg1 (orf19.4056) function.

Our results indicate that the network of genetic interactions controlling filament length varies dramatically depending on whether the cells are adherent to a glass surface or are suspended as planktonic cells. We cannot rule out the possibility that differences in culture medium (YETS *vs.* RMPI) contribute in some way to the reorganization of the TF network interactions. Because YETS medium does not induce planktonic cells to form true hyphae, we feel that signals specific to adherence are likely to make a significant contribution to the transcriptional program initiated during filamentation. Even if these changes are completely due to the medium, our data provide a dramatic example of how external cues reorganzie the specific interactions between a common set of TFs to yield a common morphological outcome.

### The network of TF interactions regulating the ratio of yeast to filaments Under planktonic conditions is similar to that for planktonic filament length

Although the relative proportion of yeast and filament forms could not be used to assess filamentation under adherent conditions for technical reasons, the Image Stream technology allowed us to examine this phenotype under planktonic conditions (Figure 8A). As mentioned above, 90% of the reference strain SN250 formed filaments. Of the heterozygous mutants, *brg1*Δ (orf19.4056) was the only strain that showed a statistically significant reduction in the proportion of filamentous cells (78% hyphae, *P* < 0.0001, Fisher’s exact test, N = 2720). Similar to the interactions observed for filament length, deletion of one allele of *ROB1* (orf19.4998), *BCR1* (orf19.723), and *EFG1* (orf19.610) in the *brg1*Δ (orf19.4056) background suppressed its haploinsufficiency and restored the *brg1*Δ double mutants to reference strain levels of filament formation (Figure 8B). The full set of data are provided in Table S6.

Consistent with the filament length data, *ROB1* (orf19.4998) showed aggravating interactions with *TEC1* (orf19.5908), *EFG1* (orf19.610), *BCR1* (orf19.723), and *NDT80* (orf19.2119) (Figure 8B&C). *TEC1* (orf19.5908) and *EFG1* (orf19.610) also show an aggravating interaction. The most severely affected double mutant is the *rob1*Δ *tec1Δ* strain (60% hyphae, *P* < 0.0001, Fisher’s exact test, N = 2468). The interaction network for aggravating interactions for this set of TFs is shown in Figure 8C. *ROB1* (orf19.4998) appears to function as a local node and as a component of a three TF module with *EFG1* (orf19.610) and *TEC1* (orf19.5908). The most common function of three-component TF modules is as feed-forward loops (Mangan and Alon 2003; Zhang *et al.* 2005). Consistent with that formulation, Efg1 (orf19.610) is known to directly bind and regulate the expression of *TEC1* (orf19.5908). Nobile *et al.* has shown that Tec1 (orf19.5908) and Efg1 (orf19.610) bind to the promoter of *ROB1* (orf19.4998) (Nobile *et al.* 2012) and, in other work, our laboratory found that Tec1 (orf19.5908) and Efg1 (orf19.610) cooperate with Rob1 (orf19.4998) to regulate *ROB1* (orf19.4998) expression (Glazier *et al.* 2017). Since all three of these TFs regulate genes that are involved in hyphae morphogenesis, it is likely that cooperative effects on target gene expression also contribute to the observed genetic interactions as well. The similarity in genetic interactions observed for filament length and proportion of filamentous cells during planktonic conditions indicates that these two phenotypes are controlled by very similar transcriptional responses.

## Discussion

The genetic analysis of simple and complex heterozygotes can provide information that is complementary to that obtained by the analysis of homozygous deletion strains. In addition, genetic interaction analysis can be problematic in diploid organisms such as *C. albicans*, particularly if the gene of interest is essential or if the homozygous deletion mutant has a very strong phenotype. Here, we described a set of strains and plasmids designed to facilitate genetic interaction analysis in *C.albicans* using CHI. We have characterized phenotypes for this set of TF heterozygous deletion strains both *in vitro* and *in vivo* as a benchmark for subsequent studies. Consistent with previous studies of the prevalence of haploinsufficiency in other yeast, the phenotype is relatively uncommon and we identified only a handful of heterozygous strains that displayed altered function.

The *in vitro* phenotype with the most haploinsufficient/proficient mutants was filamentation. Homann *et al.* found that eight homozygous TF deletion mutants were deficient for filamentation on Spider medium at 37° while an additional eight strains were hyperfilamentous (Homann *et al.* 2009). Of the eight homozygous mutants that showed decreased filamentation, three were also haploinsufficient. In contrast, all of the heterozygous counterparts of hyperfilamentous homozygous TF deletions were also hyperfilamentous. In addition, a ninth mutant, *ZFU3* (orf19.6888) showed increased filamentation. Although this strain was not hyperfilamentous in the data set reported by Homann *et al.* Vandeputte *et al.* reported that *zfu3*ΔΔ (orf19.6888) was hyperfilamentous (Homann *et al.* 2009; Vandeputte *et al.* 2011). The rate of haploinsufficiency among the negative transcriptional regulators of filamentation (100%) is strikingly above the rate for the overall genome and for other phenotypes that we tested with this set of mutants. Based on these data, it seems that *C. albicans* may be more sensitive to alterations in the gene dosage of negative regulators of hyphal morphogenesis than of positive regulators.

We did not observe haploinsufficient growth phenotypes for the TF heterozygous deletion strains on solid agar plates with rich media and only one heterozygous deletion mutant, *UGA33* (orf19.7317), showed increased growth in competitive pooled assays in the same media. Homan *et al.* found that nine homozygous mutants had growth defects under the same conditions and, therefore, it is not surprising that no haploinsufficiency was observed given the low rate of its occurrence overall (Homann *et al.* 2009). In contrast, haploinsufficient strains were identified at either 24 or 48 hr during disseminated candidiasis in a mouse model. Although there have been previous competitive infectivity screens using homozygous deletion mutants, none had used the early time points that we employed. Nearly one-half of the TF heterozygotes with altered infectivity at early time points were mutants that had been previously shown to be involved in abiotic adhesion under flow conditions (Finkel *et al.* 2012). Although we have not determined whether these strains are also haploinsufficient for adherence to abiotic surfaces under flow conditions, it is reasonable that our experiment would identify such mutants since endothelial cell attachment under conditions of flow is a crucial and early step in the establishment of a disseminated infection.

We used a set of double heterozygous mutants derived from TFs previously shown to regulate biofilm formation to genetically characterize two early steps in the establishment *C. albicans* biofilm (Nobile *et al.* 2012; Fox *et al.* 2015): adherence to an abiotic surface and filamentation. Previously, we had used the same set of double mutants to study the genetic interactions at the point when a relatively mature biofilm had formed, *i.e.*, 48 hr (Glazier *et al.* 2017). At the stage of initial adherence, we found that the set of six TFs displayed a very different set of intra-network genetic interactions relative those observed for mature biofilms. Thus, the roles of the TFs vary depending on the specific stage of biofilm formation. This is consistent with the recently reported homozygous deletion study of the same set of mutants (Fox *et al.* 2015). Combined with those results, our data support a model in which different stages of biofilm formation specify different combinations of TFs for optimal function.

We also found that *TEC1* (orf19.5908) plays an important role that was not evident from analysis of the homozygous deletion mutants. Deletion of one allele of *TEC1* (orf19.5908) in the context of a heterozgous deletion mutation in any of the other TFs led to an aggravating interaction, indicating that *TEC1* (orf19.5908) functions cooperatively with the other TFs. Consequently, Tec1 appears to be required to buffer the cell against reduced gene dosage in other biofilm-related TFs during the initial adhesion step. Further supporting this role for Tec1 (orf19.5908) is the fact that overexpression of *TEC1* (orf19.5908) increases adhesion of wild type, single heterozygotes, and double heterozygotes with decreased biofilm adhesion. Tec1 (orf19.5908) is bound by and regulated by all other network TFs during biofilm formation (Nobile *et al.* 2012) as well as by itself. The genetics of its interaction with other TFs, taken together with its regulation pattern, suggest that *TEC1* (orf19.5908) is an important output of the TF network. Furthermore, it interacts with all of the other five TFs as part of three, three-module motifs (Figure 4D). Within genetic interaction networks, three module TF motifs commonly represent feed-forward loops (Mangan and Alon 2003). Thus, the structure of the adhesion network strongly supports the notion that all of the other TFs contribute to *TEC1* (orf19.5908) expression through a feed-forward mechanism. Finally, this overall model is supported by the transcriptional profiling data reported by Fox *et al.* in that *TEC1* expression is elevated in adherent cells as compared to planktonic cells grown at the same temperature (Fox *et al.* 2015).

**Figure 9:**
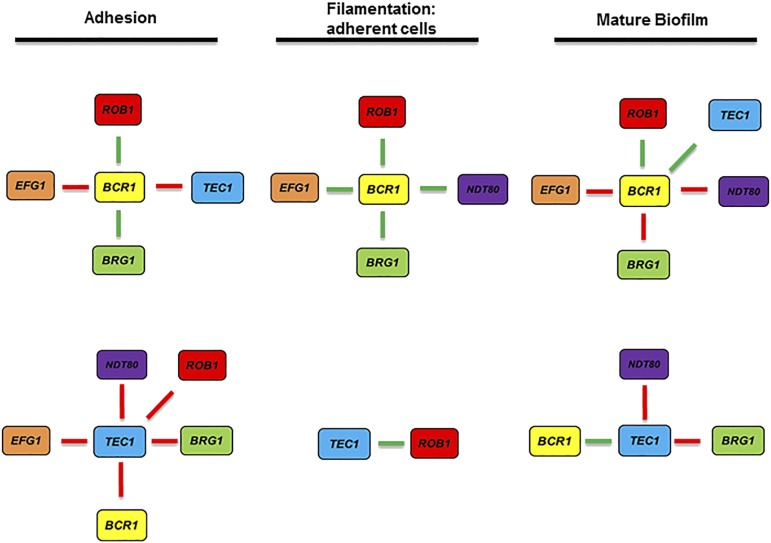
The genetic interactions of the biofilm TFs are dynamic and are temporally reorganized during biofilm formation. The genetic interaction networks for *BCR1* and *TEC1* are shown for adhesion, adherent cell filamentation, and mature biofilm stages of biofilm formation. The interaction networks for the mature biofilm stage are derived from Glazier *et al.* 2017.

One of the most potentially informative features of genetic interaction studies is its ability to provide insights into how sets of genes with common functions work together to regulate a biological process (Costanzo *et al.* 2016). Taking the genetic interactions for the biofilm-related TFs for the early stages of biofilm formation together with our previous data for the genetic interactions at a much later stage of the biofilm process (48 hr), it is clear that the network is not a static set of interactions but is instead more malleable in nature. Taking the set of Tec1 (orf19.5908) and Bcr1 (orf19.723) interactions as illustrative examples (Figure 9), the variable nature of their interactions with other network TF over the course of biofilm formation becomes quite clear. At first glance, it may seem that the interactions of *BCR1* (orf19.723) are relatively stable because it interacts with most of the TFs at each step of the process. However, only one of its thirteen interactions, an alleviating interaction with *BCR1* (orf19.723), is consistently maintained across each stage of biofilm formation. The interactions displayed *TEC1* (orf19.5908) are drastically different at each stage and none are consistently manifest at each stage. These dynamic reorganizations are characteristic of a temporal network, as opposed to a static network (Li *et al.* 2017). One of the advantages of temporal networks is that they are able to control a wide range of functions with a relatively small number of nodes (Li *et al.* 2017).

The significant changes in biofilm TF network topology and function also fit well with Luscombe *et al.* who showed that, in *S. cerevisiae*, TFs do not necessarily serve as permanent hubs but instead frequently had transient roles leading to significant changes in network topology in response, in particular, to environmental cues (Luscombe *et al.* 2004). During biofilm formation, the cell’s microenvironment alters dramatically as the the process progresses and likely provides the external cues leading to the reorganization of the network topology and the specific alterations in the roles of the specific TFs within the network. Consequently, the genetic interactions of the *C. albicans* biofilm TFs provide insights into how a relatively small set of TFs is able to orchestrate the multistep process of constructing a biofilm. What remains to be explored are the mechanisms by which the cell is able to reorganize the specific contributions of the individual TFs to execute each step of this complex process.

In summary, we have constructed a set of strains and plasmids for the systematic genetic interaction analysis of *C. albicans* TFs based on simple and complex haploinsufficiency. In addition to providing these tools and baseline phenotypic characterization for the strains, we have used the library to probe genetics regulation of the early stages of biofilm formation and filamentation. The latter analyses provide genetic support for the notion that the TF interaction networks are quite plastic with specific combinations of TFs working together under specific conditions.

## Supplementary Material

Supplemental Material is available online at www.g3journal.org/lookup/suppl/doi:10.1534/g3.117.300515/-/DC1.
